# Origin and Distribution of Thiophenes and Furans in Gas Discharges from Active Volcanoes and Geothermal Systems

**DOI:** 10.3390/ijms11041434

**Published:** 2010-03-31

**Authors:** Franco Tassi, Giordano Montegrossi, Francesco Capecchiacci, Orlando Vaselli

**Affiliations:** 1 Department of Earth Sciences, University of Florence, Via G. La Pira, 4, 50121 Florence, Italy; E-Mails: francesco.capecchiacci@unifi.it (F.C.); orlando.vaselli@unifi.it (O.V.); 2 CNR-IGG Institute of Geosciences and Earth Resources, Via G. La Pira, 4, 50121 Florence, Italy; E-Mail: montegrossi@igg.cnr.it (G.M.)

**Keywords:** heteroaromatics, geothermal fluids, volcanic fluids, furans, thiophenes

## Abstract

The composition of non-methane organic volatile compounds (VOCs) determined in 139 thermal gas discharges from 18 different geothermal and volcanic systems in Italy and Latin America, consists of C_2_–C_20_ species pertaining to the alkanes, alkenes, aromatics and *O*-, *S*- and *N*-bearing classes of compounds. Thiophenes and mono-aromatics, especially the methylated species, are strongly enriched in fluids emissions related to hydrothermal systems. Addition of hydrogen sulphide to dienes and electrophilic methylation involving halogenated radicals may be invoked for the formation of these species. On the contrary, the formation of furans, with the only exception of C_4_H_8_O, seems to be favoured at oxidizing conditions and relatively high temperatures, although mechanisms similar to those hypothesized for the production of thiophenes can be suggested. Such thermodynamic features are typical of fluid reservoirs feeding high-temperature thermal discharges of volcanoes characterised by strong degassing activity, which are likely affected by conspicuous contribution from a magmatic source. The composition of heteroaromatics in fluids naturally discharged from active volcanoes and geothermal areas can then be considered largely dependent on the interplay between hydrothermal *vs.* magmatic contributions. This implies that they can be used as useful geochemical tools to be successfully applied in both volcanic monitoring and geothermal prospection.

## 1. Introduction

Chemical constituents released from gas discharges in active and quiescent volcanic complexes and geothermal systems can be related to: 1) primary (magma degassing) and 2) secondary (gas-water-rock interactions occurring at relatively shallow depth) sources. The ratio between magmatic *vs.* hydrothermal contributions is generally indicative of the state of activity of a volcanic system and is a basic parameter in terms of volcanic surveillance [1–4]. Magma degassing produces highly acidic and corrosive gas compounds that may affect the geothermal potential of a hydrothermal reservoir. Hydrothermal fluid composition is constituted by water vapor and CO_2_ and show significant concentrations of reduced gas species, such as H_2_S, H_2_ CO and CH_4_ [5,6]. Magmatic-related fluid contributions, although mainly consisting of the same gases dominating hydrothermal fluids, *i.e.,* water vapor and CO_2_, can unequivocally be recognized in thermal discharges by the presence of highly acidic compounds, especially SO_2_ [7–9]. Secondary interactions, such as gas scrubbing processes within shallow aquifers [10,11], are able to strongly affect this highly soluble and reactive gas compounds, frequently masking any clue of magmatic-related fluid contribution at surface. The behaviour of hydrocarbons in natural fluid discharges has recently been considered as a potential tool to investigate the thermodynamic conditions controlling fluid reservoirs feeding fumarolic exhalations in volcanic and geothermal systems [12–15]. These investigations have demonstrated that light hydrocarbons, especially the C_2_–C_4_ alkenes-alkanes pairs, play an important role in both geochemical surveillance of volcanic systems and geothermal prospection. On the contrary, little attention was devoted to heavier organic compounds for similar purposes.

In the present work, 139 gas emissions from active volcanoes and geothermal systems set in different geodynamical environments were analysed for the determination of non-methane VOC (Volatile Organic Compound) composition, especially that of heteroaromatic and aromatic compounds. On the basis of this dataset, the main goals were to: 1) assess the origin of thiophenes and furans in naturally discharged fluids and 2) evaluate the possible use of these compounds as geochemical tracers to discriminate different fluid source regions in volcanic-hydrothermal environment.

## 2. Results and Discussion

### 2.1. Chemical Composition of the Main Gas Species

A representative composition of the main gas components in fluid discharges from the volcanic and geothermal systems investigated in the present study is listed in Table 1. Gas concentrations are expressed in μmol/mol and referred to the dry gas phase. Gas samples from Teide (Spain), Turrialba (Costa Rica), Vulcano (Italy), Lascar and Tacora (Chile) volcanoes have outlet temperatures varying in a wide range (from 72 to 405 °C) and show a chemical composition dominated by CO_2_ and characterised by variable amounts of SO_2_ (from 1.2 to 569394 μmol/mol).

Such features, coupled with relatively low concentrations of CH_4_ (<690 μmol/mol) and high concentrations of HCl (from 351 to 74540 μmol/mol) and H_2_ (up to 32591 μmol/mol), indicate that the gas chemistry of these systems is strongly controlled by magma degassing [16]. This hypothesis is in agreement with previous studies [17–20] that investigated the source of fluids produced by the intense fumarolic activity recently observed at these volcanic systems. A different chemistry characterises gases from (1) El Tatio (Chile), Larderello (Italy) and Tendaho (Ethiopia) geothermal systems [21–23] (samples #48–64), and (2) volcanoes whose degassing activity is considered to be mainly related to boiling of extended hydrothermal reservoirs (Copahue, Argentina; Deception, Antarctica; El Chichon, Mexico; Ischia, Pantelleria, Phlegrean Fields and Vesuvio, Italy; Nisyros, Greece; Tatun, Taiwan; Yellowstone, USA) [24–31] (samples #65–139). This group shows SO_2_ below the instrumental detection limit (≈0.01μmol/mol: samples #48–138), relatively low outlet temperatures <118 °C, high CH_4_ concentrations (up to 64103 μmol/mol), and HCl not exceeding 500 μmol/mol.

### 2.2. VOC Composition

**Figure 1.**
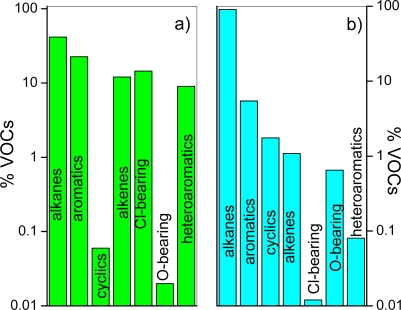
Relative concentrations, expressed as % of the total VOC abundances, of alkane, aromatic, cyclic, alkene, *Cl*-bearing, *O*-bearing and heteroaromatic compounds in (**a**) *M* and (**b**) *H* gases.

Up to 129 different non-methane VOCs, pertaining to the alkane (27 compounds), aromatic (21 compounds), cyclic (17 compounds), alkene (15 compounds), *Cl*-bearing (13 compounds), *O*-bearing (ketones, aldehydes, organic acids and alcohols; 36 compounds) and heteroaromatic (7 compounds) groups, were determined (Table 2; gas concentrations are expressed in ppb by volume and referred to the dry gas phase). The total VOC concentrations in gases with a dominating magmatic contribution (samples #1–47, hereafter *M* gases) are relatively low (from 61 to 1664 ppbv), whereas they range from 180 to 1235942 ppbv (Table 2) in those gases (#48–141) characterised by prevalent hydrothermal contribution (hereafter *H* gases). Hydrothermal reservoirs, even when associated with volcanic systems, are commonly recharged by fluids circulating within organic-bearing sedimentary rocks. This organic source is then transformed into VOCs through biogenic and thermogenic processes [32]. Therefore, relatively high VOC concentrations in the *H* gases are expected. On the contrary, the organic-rich component constitutes a minor fraction of the *M* gases, since it is generally destroyed by high-temperature, oxidizing fluids released from the magmatic melts [13]. The relative percentages (mean values) of the different groups of VOCs can provide preliminary indications to distinguish the *M* and *H* gases: in the *M* gases, alkane, *Cl*-bearing, aromatic, heteroaromatic and alkene compounds are present in almost comparable amounts (31, 28, 16, 16 and 9% of total VOCs, respectively), whereas cyclic and *O*-bearing species represent a small VOC fraction (<0.04%) (Figure 1a). The organic fraction of the *H* gases is largely dominated by alkanes and aromatics (54 and 24%, respectively) with minor cyclic, alkene, heteroaromatic and O-bearing compounds (from 0.7 to 1.4%), and traces (<0.04%) of Cl-bearing compounds (Figure 1b). These evidences are consistent with recent investigations that have highlighted a recurrent relation between VOC speciation and thermodynamic conditions at the fluid source in gas discharges from volcanic and geothermal systems [33–36]. Predominance of alkanes and aromatics in both the *M* and *H* gases was considered to reflect the proceeding of “reforming” processes, which in geothermal areas, as well as in hydrothermal systems commonly surroundings active volcanoes, are favoured by the large availability of catalytic agents, such as free acids, allumosilicates and sulphur gas species [37,38]. Pyrolysis of organic material was found to produce mostly alkanes and, secondarily, aromatics [39,40], whereas Fischer-Tropsch reactions were invoked for the production of light alkanes and, at a minor extent, alkenes [41]. The presence of halocarbons in volcanic gas emissions was attributed to either the product of pyrolysis of adjacent vegetation [42,43] or, alternatively, air contamination [44,45]. Conversely, organic geochemical evidence supported a pristine abiogenic origin by high-temperature gas-phase radical reactions [46,47].

### 2.3. Distribution and Origin of the Heteroaromatic Compounds

**Figure 2.**
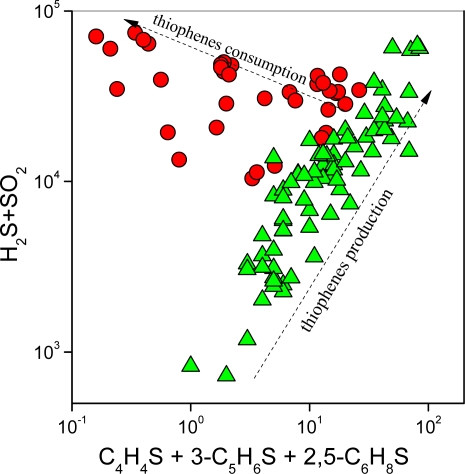
(H_2_S + SO_2_) *vs.* **(**C_4_H_4_S + 3C_5_H_6_S + 2,4C_6_H_8_S) binary diagram. Green triangle: *H* gas; red circle: *M* gas.

Concentrations (in ppbv, referred to the dry gas phase) of C_4_H_4_O, 3-C_5_H_6_O, C_4_H_8_O, 2-C_5_H_10_O, C_4_H_4_S, 3-C_5_H_6_S and 2,5-C_6_H_8_S, and those of the simplest aromatics (C_6_H_6_ and C_7_H_8_), are reported in Table 3. In the M gases, the concentrations of furans tend to be higher than those of thiophenes (their sum ranging from 1.9 to 35 and from 0.2 to 26 ppbv, respectively); C_4_H_4_S is largely the most abundant S-bearing compound (up to 1.9 ppbv), whereas C_4_H_4_O (up to 31 ppbv) dominates the furan composition. Conversely, the H gases have relatively high concentrations of C_4_H_4_S and 3-C_5_H_6_S (up to 191 and 121 ppbv, respectively), minor 2,4-C_6_H_8_S (up to 13 ppbv), and no furans, with the only exception of those from Deception, Nisyros, Vesuvio, Copahue and El Chichon volcanoes. As shown in Figure 2, in the H gases thiophenes are strongly related to H_2_S, (in hydrothermal environment SO_2_ concentrations are the below detection limit; Table 1). This correlation would imply that the formation of the S-bearing heteroaromatics intimately depends on sulphur fugacity (fS) at the fluid source, and likely occurs within deep fluid reservoirs where H_2_S is also produced. This hypothesis is consistent with the composition of fluids from carbonate reservoirs affected by thermochemical sulphate reduction: the higher the H_2_S fugacity, the higher content of organic sulphur compounds in the coexisting hydrocarbon phase [48,49]. Moreover, sulphidation of organic matter giving rise to thiophenes was found to be associated with gold mineralization deriving from hydrothermal fluids [50,51]. According to these considerations, it is reasonable to suppose that thiophenes can efficiently be produced in a hydrothermal reservoir, this environment being commonly characterised by reducing conditions, relatively high fS and temperature <350 °C [5,6]. Production of C_4_H_4_S by reaction of light alkenes, such as C_2_H_4_, with FeS_2_ and H_2_S was invoked to explain their presence in the volcanic gases emitted from Mt. Etna [52].

**Scheme 1.**
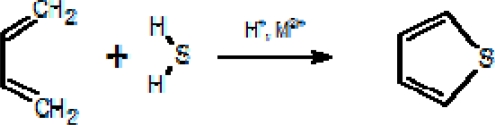
Synthesis of C_4_H_4_S from butadiene.

On an industrial scale thiophene is synthesized through the following catalytic processes: 1) reaction of C_4_^+^ alcohols or carbonyls with CS_2_ over alkali-promoted alumina; 2) reaction of unsaturated aldehydes with H_2_S over an alkali-promoted alumina; 3) reaction of C_4_^+^ alkyl hydrocarbons or olefins with CS_2_, S, and H_2_S over alkali-promoted alumina; 4) catalytic dehydrogenation of tetrahydrothiophene; 5) synthesis from furan and H_2_S over alumina [53–56]. The thiophene Paal-Knorr synthesis involves the reaction of 1,4-diketones with H_2_S as sulphurising agent [57]. Generally speaking, in gases from natural fluid discharges, the most reliable genetic mechanism for the formation of thiophene is through the addition of H_2_S to dienes in the presence of H^+^ and metal catalysts (Scheme 1).

In the *M* gases heteroaromatics and inorganic sulphur-bearing gases, the latter being constituted by SO_2_ and H_2_S at comparable concentrations (Table 1), are apparently showing an inverse correlation (Figure 2). This suggests that thiophenes, which are less reactive than other five-membered heteroaromatics, including furans, serving as dienes during Diels-Alder reactions [58], tend to be destroyed when fluid reservoirs are affected by conspicuous contribution from magmatic degassing.

**Figure 3.**
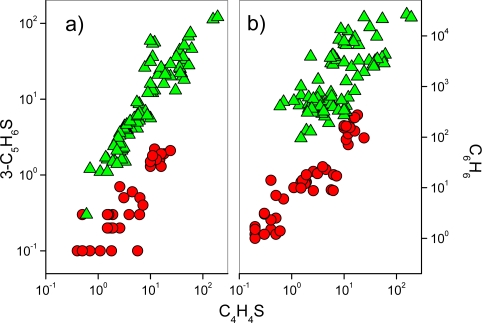
(**a**) 3-C_5_H_6_S *vs.* C_4_H_4_S and (**b**) C_6_H_6_ *vs.* C_4_H_4_S binary diagrams. Symbols as in Figure 2.

The C_4_H_4_S concentrations and those of 3-C_5_H_6_S (Figure 3a) and C_6_H_6_ (Figure 3b) show a positive correlation in both *H* and *M* gases. This supports the following hypotheses: 1) at hydrothermal conditions mono-aromatics and thiophenes are efficiently produced by similar genetic processes; 2) all these compounds have a similar behaviour in response to thermodynamic conditions caused by presence of oxidizing and high temperature (>400 °C) magmatic fluids.

**Figure 4.**
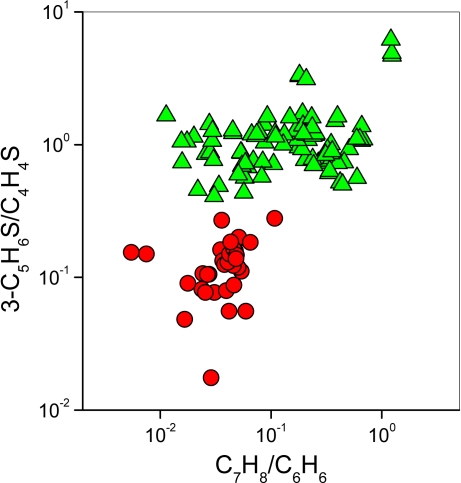
3-C_5_H_6_S/C_4_H_4_S *vs.* C_7_H_8_/C_6_H_6_ binary diagram. Symbols as in Figure 2.

**Scheme 2.**
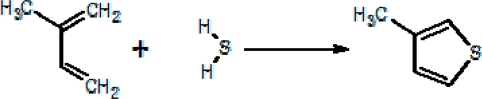
3-Methylthiophene production from 2-methylbutadiene.

It is worth noting that the *H* gases have higher 3-C_5_H_6_S/C_4_H_4_S and C_7_H_8_/C_6_H_6_ ratios than the *M* ones (Figure 4). This may be caused by the large availability of CH_4_ (Table 1) and light hydrocarbons (Table 2) that at hydrothermal conditions can produce free and halogenated radicals that favour the production of 3-C_5_H_6_S from C_4_H_4_S, as well as that of C_7_H_8_ from C_6_H_6_. Attach of XCH_3_^+^ (X = F or Cl), whose formation likely occurs in both geothermal and volcanic fluid reservoirs where halogenated species are abundant [6–9], on thiophene may give rise to the corresponding methylated derivatives [59]. It is worthy of noting that 3-C_4_H_4_S is the only methyl-thiophene recognized in both geothermal and volcanic gases (Table 3), although electrophilic methylation of thiophene is able to produce different isomers. This may be explained by the occurrence of secondary isomerization of methylated thiophenes favouring 3-C_4_H_4_S that results the thermodynamically most stable isomer in natural environments. Alternatively, 3-C_4_H_4_S may be produced through H_2_S adding to dienes, such as 2-methylbutadiene originated by isomerization of 1.3-pentadiene (Scheme 2). Double methylation seems to be favoured when methyl substitutions are stabilized at positions 2 and 4 (Table 3).

**Figure 5.**
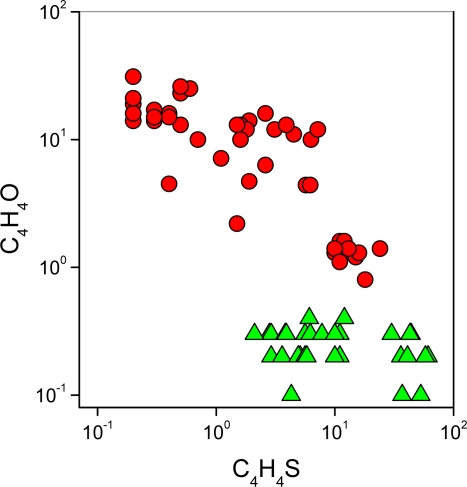
C_4_H_4_O *vs.* C_4_H_4_S binary diagram. Symbols as in Figure 2.

In the *M* gases, C_4_H_4_O is inversely correlated to C_4_H_4_S (Figure 5). This suggests that the production of C_4_H_4_O is particularly efficient in a magmatic-related environment, where thermodynamic conditions promote the destruction of thiophenes and aromatics.

**Scheme 3.**
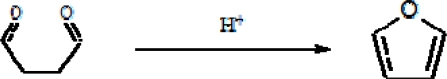
Paal-Knorr synthesis of C_4_H_4_O.

The main mechanism of formation of C_4_H_4_O may be related to the Paal-Knorr synthesis (Scheme 3), which is efficient under acidic conditions, such as those determined by the huge amounts of highly acidic gas species (HF, HCl and SO_2_) occurring in the *M* gases (Table 1).

**Figure 6.**
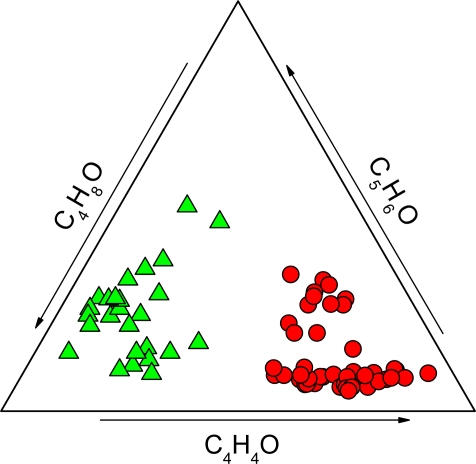
C_4_H_4_O-C_5_H_6_O-C_4_H_8_O triangular diagram. Symbols as in Figure 2.

**Scheme 4.**
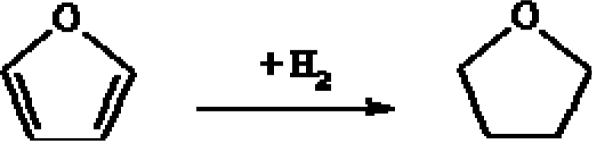
Furan hydrogenation to form C_4_H_8_O.

As shown in Figure 6, the *H* and *M* gases can also be clearly distinguished on the basis of the relative concentrations of furans: C_4_H_8_O is dominant in the *H* gases, whereas C_4_H_4_O is the most abundant O-bearing heteroaromatic species in the *M* gases. This suggests that reducing conditions and relatively low temperature (<350 °C), typical of hydrothermal environments, tend to favour the consumption of C_4_H_4_O to produce C_4_H_8_O through catalytic hydrogenation (Scheme 4) [60,61].

## 3. Experimental Section

### 3.1. Gas Sampling Method

Gas samples for the determination of the main gas species were collected into pre-evacuated 60 mL glass flasks filled with 20 mL of a 4N NaOH and 0.15 M Cd(OH)_2_ suspension. Quartz-glass dewar tubes and a plastic funnel were used to convoy the gas into the sampling flasks from 1) fumarolic vents and 2) boiling pools, respectively. During sampling, CO_2_, SO_2_ and HCl dissolved into the alkaline solution, water vapour condensed, and H_2_S reacted with Cd^2+^ to form insoluble CdS, allowing the residual gases (N_2_, CH_4_, Ar, O_2_, H_2_, and light hydrocarbons) to be concentrated in the head-space [62–64]. Gas samples for the determination of VOC composition were collected with the same devices used for the conventional gas sampling, and stored into pre-evacuated 12 mL glass vials equipped with pierceable rubber septum (Labco Exetainer^®^).

### 3.2. Analytical Methods

Nitrogen, Ar, O_2_ and H_2_ were analysed with a Shimadzu 15A gas-chromatograph equipped with Thermal Conductivity Detector (TCD) and a 9 m, 5A molecular sieve column. Methane and C_1_–C_4_ alkanes and alkenes were analysed with a Shimadzu 14a gas-chromatograph equipped with Flame Ionization Detector (FID) and a 10 m long stainless steel column (φ = 2 mm) packed with Chromosorb PAW 80/100 mesh coated with 23% SP 1700. The alkaline solution, separated from the solid precipitate by centrifugation at 4,000 rpm for 30 min, was used for the determination of: 1) CO_2_ as CO_3_^2−^ by titration with 0.5 N HCl solution; 2) SO_2_ as SO_4_^2−^, after oxidation with H_2_O_2_, by ion-chromatography (Metrohm Compact 761); 3) HCl, as Cl^−^ by ion-chromatography. The solid precipitate was oxidized by H_2_O_2_ to determine H_2_S as SO_4_^2−^ by ion-chromatography [63,64]. The analytical error is <5%.

The VOCs were pre-concentrated and transferred from the sampling vials into the column headspace of a Thermo Trace GC Ultra gas chromatograph by using a manual SPME (solid-phase micro-extraction) device introduced through the silicon membrane of the glass vial to expose the gas mixtures to a divinylbenzene (DVB)-Carboxen-polydimethylsiloxane (PDMS), 50/30 μm, 2 cm long fibre assembly (Supelco; Bellefonte, PA, USA) for 15 min [65]. The usefulness of the SPME method [66] for the VOC analysis has widely been demonstrated [67–69]. The DVB-Carboxen-PDMS fibre was selected by its high retentive properties, a feature that is particularly appropriate for analysis aimed to the determination of the organic compounds of interest for the present paper. A Thermo Trace GC Ultra gas chromatograph coupled with a Thermo DSQ Quadrupole Mass Spectrometer was used for analytical separation and detection. The mass spectrometer operated in full scan mode, in the mass range 40–400 *m*/*z*. The transfer-line temperature was set at 230 °C. The mass detector was equipped with EI set at 70 eV. The source temperature was 250 °C. The gas chromatograph was equipped with a split/splitless injection port operating in the splitless mode with a dedicated SPME liner (0.75 mm i.d.). Analytes were desorbed from the SPME fiber through direct exposure for 2 min in the GC injection port, heated at 230 °C. The chromatographic column was a 30 m × 0.25 mm i.d. 0.25 μm film thickness TV1-MS fused silica capillary column (Thermo). The carrier gas was helium set to a flow-rate of 1.3 mL/min in constant pressure mode. The column oven temperature program was the following: 35 °C (hold 10 min), rate 5.5 °C/min to 180 °C (hold 3 min), rate 20 °C/min to 230 °C (hold 6 min) [65]. Compounds were identified by comparison of the mass spectra with those of the NIST05 library (NIST, 2005).

The VOCs identified by mass spectrometry were quantified using an external standard calibration procedure performed on the basis of calibration curves created by analyzing gaseous standard mixtures of the main VOC groups, *i.e.,* alkanes, alkenes, aromatics, cyclics, chlorofluorocarbons, ketones, aldehydes and heteroaromatics. The values of the Relative Standard Deviation (RDS), calculated from seven replicate analyses of a gaseous mixture in which the compounds of interest were present at a concentration of 2 ppmv, are <7%. Eventually, the detection limits were determined by linear extrapolation from the lowest standard in the calibration curve using the area of a peak having a signal/noise ratio of 5 [68].

## 4. Conclusions

The distribution of thiophenes and furans in gases from hydrothermal and magmatic-hydrothermal systems have been revealed to be strongly dependent on the physical-chemical conditions acting on fluid reservoirs, where VOCs are produced via a complex series of catalytic processes, involving organic matter buried in sedimentary formations. Thiophene seems to be efficiently produced at hydrothermal conditions and tend to be destroyed in presence of hot, highly oxidizing fluids from a magma source. On the contrary, the formation of C_4_H_4_O seems to be favoured at highly acidic and oxidizing conditions that are determined by the presence of fluids from magmatic degassing. Methylated and hydrogenated heteroaromatics are also preferentially associated with hydrothermal conditions. According to these considerations, the composition of O- and S-bearing heteroaromatics can be utilized in both volcanic and geothermal systems to evaluate contributions of fluids produced in different “natural dominions”, *i.e.,* hydrothermal and magmatic. These results may imply useful applications in volcanic monitoring and geothermal prospection, although the existing dataset should be expanded to better constrain the behaviour of these new geochemical tracers. Experimental runs, able to test the mechanisms of formation and stability of heteroaromatics at temperature, redox and catalytic conditions resembling those of a volcano-hydrothermal environment, would probably useful to better constrain their behaviour.

## Figures and Tables

**Table 1.** Chemical composition of the main gas species. Concentrations are in μmol/mol.

**n°**	**sample**	**Location**	**T °C**	**CO**_**2**_	**HCl**	**SO**_**2**_	**H**_**2**_**S**	**N**_**2**_	**CH**_**4**_	**Ar**	**O**_**2**_	**H**_**2**_
1	Teide volcano 1	Spain	98	960092	351	1502	17807	15444	15	218	688	4191
2	Teide volcano 2	Spain	96	968658	469	1203	16838	7700	22	82	421	5044
3	Lascar volcano 1	Chile	76	753737	5739	5995	4440	207209	488	1293	464	19269
4	Lascar volcano 2	Chile	80	784999	5061	6526	4796	179060	734	1225	461	15917
5	Lascar volcano 3	Chile	76	755275	8570	7078	5264	200992	689	1416	557	18226
6	Lascar volcano 4	Chile	72	743534	14702	20438	10298	186298	431	983	597	20241
7	Lascar volcano 5	Chile	73	768092	29506	24890	8596	144002	267	332	285	20683
8	Lascar volcano 6	Chile	82	765675	13670	21603	8180	167442	225	259	770	20551
9	Lascar volcano 7	Chile	151	834908	16522	46074	1977	81114	53	89	622	16117
10	Lascar volcano 8	Chile	178	911092	6045	27092	1540	36634	29	60	530	6655
11	Lascar volcano 9	Chile	250	855579	12513	48617	1514	67155	17	78	654	12146
12	Lascar volcano 10	Chile	154	841254	12698	42327	1817	92577	45	91	677	6286
13	Lascar volcano 11	Chile	174	840461	14185	46693	1986	84126	51	134	512	8920
14	Lascar volcano 12	Chile	150	853908	12795	44835	1840	74669	44	132	485	8587
15	Tacora volcano 1	Chile	84	852515	1093	6216	26797	111840	222	124	12	873
16	Tacora volcano 2	Chile	84	874379	683	3237	25178	94964	273	79	137	857
17	Tacora volcano 3	Chile	84	805893	891	4708	28855	156479	289	155	810	1544
18	Tacora volcano 4	Chile	84	950768	1141	6722	27604	13455	21	14	36	85
19	Tacora volcano 5	Chile	83	958930	756	4040	22328	13640	26	16	55	124
20	Tacora volcano 6	Chile	82	941778	945	6235	36189	14544	23	23	12	115
21	Tacora volcano 7	Chile	84	952234	1042	4899	29150	12384	31	15	26	106
22	Tacora volcano 8	Chile	84	947883	1043	6093	31066	13550	43	15	25	127
23	Tacora volcano 9	Chile	91	942904	891	5731	35951	14193	34	15	25	106
24	Tacora volcano 10	Chile	90	947666	1010	4589	33215	13219	35	14	19	100
25	Turrialba volcano 1	Costa Rica	91	914529	6694	103729	69036	9424	2.4	8.7	3.8	302
26	Turrialba volcano 2	Costa Rica	90	907636	4354	69829	73642	14233	3.6	16	64	50
27	Turrialba volcano 3	Costa Rica	91	966244	14796	569394	17254	540	2.5	0.4	2.2	1163
28	Turrialba volcano 4	Costa Rica	93	963564	7214	407465	20074	5507	2.9	6.9	5.8	3628
29	Vulcano Island crater 1	Italy	311	930405	31870	213218	8307	27345	1.5	42	1679	336
30	Vulcano Island crater 2	Italy	317	933226	39026	107870	8527	16263	0.6	18	1044	1895
31	Vulcano Island crater 3	Italy	316	880815	74540	370065	15292	26865	0.9	36	1506	945
32	Vulcano Island crater 4	Italy	236	951272	6184	87863	25117	8534	0.7	15	13	8866
33	Vulcano Island crater 5	Italy	208	968131	15517	55466	4584	11112	0.5	14	0.9	642
34	Vulcano Island crater 6	Italy	102	992971	275	9846	3580	3083	0.6	2.3	0.5	88
35	Vulcano Island crater 7	Italy	278	952908	21738	29433	5395	16839	0.4	62	748	2310
36	Vulcano Island crater 8	Italy	102	933102	604	21475	20917	44602	2.2	136	226	411
37	Vulcano Island crater 9	Italy	251	967834	10141	29045	10562	10901	1.0	10	3.4	549
38	Vulcano Island crater 10	Italy	209	915616	11881	43365	20983	40038	1.3	37	140	11304
39	Vulcano Island crater 11	Italy	405	949811	21110	54086	16790	11306	0.4	10	25	950
40	Vulcano Island crater 12	Italy	245	927050	37358	126458	13795	17316	3.3	12	2.8	4461
41	Vulcano Island crater 13	Italy	295	920470	33885	87013	31162	12904	0.7	17	6.3	1556
42	Vulcano Island crater 14	Italy	215	950604	19154	70022	4480	17052	1.6	24	1381	7304
43	Vulcano Island crater 15	Italy	289	921121	27023	105545	23028	20213	5.1	36	95	8479
44	Vulcano Island crater 16	Italy	390	981526	1886	12977	6404	9060	0.4	9	31	1083
45	Vulcano Island crater 17	Italy	101	968938	2805	12052	8632	19343	0.3	17	116	150
46	Vulcano Island crater 18	Italy	213	951697	2010	47143	20640	7412	1.0	19	33	18187
47	Vulcano Island crater 19	Italy	325	848331	54387	132552	36150	25940	4.6	31	2564	32591
48	El Tatio 1	Chile	86	989864			3285	6548	116	42	32	114
49	El Tatio 2	Chile	84	989707			3669	6278	77	47	25	196
50	El Tatio 3	Chile	86	993055			2020	4483	197	41	59	144
51	El Tatio 4	Chile	87	992536			1181	5797	219	55	57	155
52	El Tatio 5	Chile	84	993204			725	5472	416	50	46	87
53	Afar 1	Ethiopia	99	972419	59		3618	18229	1210	443	3929	8.8
54	Afar 2	Ethiopia	99	973510	77		10824	8094	1152	186	3475	2416
55	Afar 3	Ethiopia	96	973831	75		12280	7526	1407	181	2295	2386
56	Afar 4	Ethiopia	97	959562	49		12484	20489	1156	505	5718	6.2
57	Afar 5	Ethiopia	98	970847	78		14365	7220	2029	163	2477	2680
58	Afar 6	Ethiopia	92	965397	37		12540	15743	598	383	5200	27
59	Afar 7	Ethiopia	97	975181	32		11710	9043	900	222	2877	10
60	Larderello 1	Italy	93	924663			25129	15053	14458	53	279	20364
61	Larderello 2	Italy	95	736044			6762	194995	4252	2734	46147	9066
62	Larderello 3	Italy	90	936040			13026	13887	9018	59	279	27691
63	Larderello 4	Italy	91	834318			8977	102383	4056	1465	27642	21158
64	Larderello 5	Italy	85	913603			18270	15737	24420	88	151	27881
65	Deception Island 1	Antarctica	99	984350			5127	8196	16	180	1454	678
66	Deception Island 2	Antarctica	98	983391			5910	8373	25	177	2027	120
67	Deception Island 3	Antarctica	99	986079			6042	5712	50	123	499	1495
68	Copahue volcano 1	Argentina	93	960692	542		10433	14522	6443	55	194	7120
69	Copahue volcano 2	Argentina	90	958455	19		9900	14557	6484	49	120	10416
70	Copahue volcano 3	Argentina	85	979580	18		5392	6944	3687	22	70	4288
71	Copahue volcano 4	Argentina	92	969720	678		11394	6684	5762	28	188	5546
72	Copahue volcano 5	Argentina	75	988659	5.3		2483	2701	3659	10	25	2457
73	Copahue volcano 6	Argentina	91	972792	476		7785	8707	3966	26	87	6162
74	Copahue volcano 7	Argentina	93	984796	438		14106	295	185	3.2	10	168
75	Copahue volcano 8	Argentina	92	960472	587		17743	3802	682	18	232	16465
76	Copahue volcano 9	Argentina	80	938355	545		10171	25914	10270	497	3488	10761
77	Copahue volcano 10	Argentina	92	934503	393		20052	12769	26963	61	232	5026
78	Nysiros Island 1	Greece	99	773472	15		201369	2581	11896	8.8	9.4	10649
79	Nysiros Island 2	Greece	103	867419	8.6		131034	174	378	0.4	0.8	985
80	Nysiros Island 3	Greece	104	847257	7.2		136449	1930	7563	10	14	6771
81	Nysiros Island 4	Greece	100	820625	12		173199	779	2169	3.8	9.1	3203
82	Nysiros Island 5	Greece	98	827560	8.3		165943	582	2300	1.4	2.0	3603
83	Nysiros Island 6	Greece	101	825192	7.0		151180	4058	545	22	40	18957
84	Nysiros Island 7	Greece	102	732743	8.3		169548	13036	31187	44	90	53344
85	Nysiros Island 8	Greece	97	781709	7.0		162342	2792	25678	11	15	27446
86	Nysiros Island 9	Greece	98	821809	5.8		176132	724	200	2.4	6.5	1120
87	Nysiros Island 10	Greece	100	778698	12		210844	582	1845	2.0	0.7	8017
88	Nysiros Island 11	Greece	101	821130	9.2		143847	10159	10511	33	288	14023
89	Nysiros Island 12	Greece	102	895023	5.9		60765	11314	22953	34	198	9707
90	Nysiros Island 13	Greece	98	822764	10		160681	10344	2752	38	4.7	3407
91	Nysiros Island 14	Greece	100	827067	5.8		170324	226	124	0.7	1.3	2251
92	Ischia Island 1	Italy	101	913479			2259	66747	20	262	17067	167
93	Ischia Island 2	Italy	102	960463			2431	26029	21	97	10756	202
94	Ischia Island 3	Italy	99	986746			2724	9154	121	34	675	546
95	Ischia Island 4	Italy	96	981471			5166	5555	52	25	268	7463
96	Ischia Island 5	Italy	101	918959			3957	58294	17	245	18317	211
97	Phlegrean Fields 1	Italy	59	995375			2731	1159	31	6.1	31	667
98	Phlegrean Fields 2	Italy	78	994106			3091	1721	59	5.2	14	1003
99	Phlegrean Fields 3	Italy	102	988558	270		8240	1817	49	5.8	5.8	1055
100	Phlegrean Fields 4	Italy	101	987451	373		8910	1802	40	8.0	73	1343
101	Phlegrean Fields 5	Italy	161	972565	440		11090	7931	146	28	17	7782
102	Phlegrean Fields 6	Italy	163	985513	165		10967	2002	30	2.9	16	1304
103	Phlegrean Fields 7	Italy	162	986765	140		9860	1843	36	3.0	7.8	1344
104	Phlegrean Fields 8	Italy	148	984348	201		10867	2397	52	4.8	32	2098
105	Phlegrean Fields 9	Italy	149	977398	238		17418	2693	54	4.3	7.4	2188
106	Phlegrean Fields 10	Italy	103	989567	105		8030	1406	25	2.9	8.7	855
107	Phlegrean Fields 11	Italy	101	982152	147		13807	2210	204	4.8	17	1459
108	Vesuvio volcano 1	Italy	89	973247			14224	4002	424	13	34	7623
109	Vesuvio volcano 2	Italy	87	975046			14545	1961	414	5.3	20	7532
110	Pantelleria Island 1	Italy	61	995172			3139	1431	185	35	38	0.1
111	Pantelleria Island 2	Italy	99	978349			3051	14268	5.0	372	3954	0.3
112	Pantelleria Island 3	Italy	101	977463			4806	1939	7360	27	55	8349
113	El Chichon volcano 1	Mexico	78	934500			17871	12636	50	81	391	34470
114	El Chichon volcano 2	Mexico	100	973559	52		15085	3394	75	8.5	2.6	7883
115	El Chichon volcano 3	Mexico	96	952598			22350	13983	41	73	495	10457
116	El Chichon volcano 4	Mexico	101	912794	91		58729	15099	230	35	174	12947
117	El Chichon volcano 5	Mexico	83	826372			14916	101241	100	354	2587	54430
118	El Chichon volcano 6	Mexico	46	925271			34423	20511	34	102	206	19452
119	Tatun 1	Taiwan	98	719901			124983	81518	64103	685	7855	955
120	Tatun 2	Taiwan	98	780717			184829	23606	10790	33	12	13
121	Tatun 3	Taiwan	118	915935			60426	16571	6681	27	60	300
122	Tatun 4	Taiwan	101	975848			2606	20744	528	120	147	6.5
123	Tatun 5	Taiwan	95	930985			62801	5085	1180	6.9	1.0	2.9
124	Tatun 6	Taiwan	91	949840			825	48445	467	111	298	14
125	Tatun 7	Taiwan	98	860849			116573	9269	11546	7.5	2.9	1753
126	Yellowstone 1	U.S.A.	93	987886			7396	1967	276	30	8	1417
127	Yellowstone 2	U.S.A.	85	959774			15997	16332	1956	414	10	5450
128	Yellowstone 3	U.S.A.	87	990440			6432	1925	369	29	2.7	736
129	Yellowstone 4	U.S.A.	91	904177			33339	2016	29971	5.0	48	19315
130	Yellowstone 5	U.S.A.	92	964894			27585	5348	443	135	19	1475
131	Yellowstone 6	U.S.A.	92	974036			22715	2449	560	47	10	157
132	Yellowstone 7	U.S.A.	92	771017			21120	176009	2865	2134	26063	111
133	Yellowstone 8	U.S.A.	93	953746			38210	3321	2962	84	11	1512
134	Yellowstone 9	U.S.A.	94	956582			19888	15984	3549	430	77	3411
135	Yellowstone 10	U.S.A.	94	965209			20181	4497	2050	107	29	7031
136	Yellowstone 11	U.S.A.	115	985033			11544	2131	411	33	9.1	793
137	Yellowstone 12	U.S.A.	93	977044			18030	2620	265	59	15	1952
138	Yellowstone 13	U.S.A.	91	966463			23989	5554	3530	135	5.0	250
139	Yellowstone 14	U.S.A.	93	935528			23596	30124	9281	785	54	79

**Table 2.** Composition of main VOC groups. Concentrations are in ppbv.

**n°**	**sample**	**Alkanes**	**Aromatics**	**Cyclics**	**Alkenes**	**Cl-Bearing**	**O-bearing**	**heteroaromatics**	**sum**
1	Teide volcano 1	400	73	0.2	219	1.8	0.18	16	710
2	Teide volcano 2	346	91	0.2	206	3.6	0.17	15	662
3	Lascar volcano 3	112	25	0.2	25	39	0.07	27	228
4	Lascar volcano 4	98	23	0.2	16	34	0.05	24	195
5	Lascar volcano 5	79	27	0.1	36	41	0.06	27	210
6	Lascar volcano 6	81	30	0.1	41	36	0.08	26	214
7	Lascar volcano 7	67	22	0.1	52	74	0.07	25	239
8	Lascar volcano 8	73	21	0.2	48	37	0.08	30	209
9	Lascar volcano 9	41	16	0.1	16	46	0.03	26	145
10	Lascar volcano 10	36	18	0.1	15	35	0.04	23	126
11	Lascar volcano 11	46	15	0.1	21	31	0.02	23	136
12	Lascar volcano 12	42	16	0.1	15	56	0.03	21	150
13	Lascar volcano 13	33	17	0.1	18	66	0.05	21	155
14	Lascar volcano 14	29	17	0.1	14	63	0.04	19	142
15	Tacora volcano 1	580	262	1.1	142	2.5	0.08	19	1007
16	Tacora volcano 2	718	305	0.9	91	3.9	0.06	22	1141
17	Tacora volcano 3	1222	267	0.8	150	4.7	0.06	20	1664
18	Tacora volcano 4	96	116	0.3	11	3.5	0.05	29	256
19	Tacora volcano 5	82	147	0.2	15	4.6	0.07	18	267
20	Tacora volcano 6	86	157	0.3	13	5.4	0.08	20	283
21	Tacora volcano 7	58	182	0.4	10	3.8	0.06	18	273
22	Tacora volcano 8	96	176	0.5	14	6.1	0.04	14	307
23	Tacora volcano 9	110	197	0.5	15	4.8	0.09	14	341
24	Tacora volcano 10	105	196	0.4	12	4.8	0.05	15	333
25	Turrialba volcano 5	11	11	0.0	7.3	29	0.02	13	71
26	Turrialba volcano 6	12	10	0.0	5.5	28	0.01	15	70
27	Turrialba volcano 7	9.0	10	0.0	6.3	31	0.02	13	69
28	Turrialba volcano 8	13	11	0.0	6.9	36	0.03	12	79
29	Vulcano Island crater 1	27	2.5	0.0	4.6	64	0.02	25	123
30	Vulcano Island crater 2	37	9.4	0.0	4.5	61	0.03	19	131
31	Vulcano Island crater 3	37	2.7	0.0	8.8	15	0.02	32	95
32	Vulcano Island crater 4	15	1.7	0.0	5.1	69	0.01	22	113
33	Vulcano Island crater 5	10	1.4	0.0	2.3	71	0.01	25	110
34	Vulcano Island crater 6	4.3	1.2	0.0	1.7	45	0.01	16	68
35	Vulcano Island crater 7	20	1.7	0.0	3.9	95	0.01	30	150
36	Vulcano Island crater 8	76	20	0.0	11	36	0.02	8.3	151
37	Vulcano Island crater 9	8.4	1.9	0.0	1.8	15	0.01	34	61
38	Vulcano Island crater 10	41	2.5	0.0	10	36	0.01	23	112
39	Vulcano Island crater 11	13	2.0	0.0	2.1	35	0.02	40	93
40	Vulcano Island crater 12	20	3.7	0.0	8.6	42	0.03	21	95
41	Vulcano Island crater 13	14	1.1	0.0	2.9	74	0.02	24	116
42	Vulcano Island crater 14	27	3.6	0.0	6.0	46	0.01	26	109
43	Vulcano Island crater 15	20	2.8	0.0	5.1	26	0.01	29	83
44	Vulcano Island crater 16	25	8.0	0.0	7.0	31	0.02	35	106
45	Vulcano Island crater 17	24	17	0.0	4.2	26	0.01	5.3	76
46	Vulcano Island crater 18	97	16	0.0	16	38	0.02	10	177
47	Vulcano Island crater 19	56	1.4	0.0	13	85	0.01	22	178
48	El Tatio 1	2104	861	7.2	19	1.5	9.1	3.2	3005
49	El Tatio 2	2712	1715	5.9	113	1.6	8.8	3.7	4560
50	El Tatio 3	1165	524	4.5	19	1.2	4.5	3.7	1721
51	El Tatio 4	1228	715	2.6	20	1.1	5.2	2.7	1975
52	El Tatio 5	1063	601	2.3	17	1.8	3.3	2.3	1691
53	Afar 1	7632	2942	242	147	1.4	120	11	11096
54	Afar 2	11067	2789	365	245	2.3	150	16	14634
55	Afar 3	19896	3514	522	276	4.1	168	15	24395
56	Afar 4	16225	4512	197	517	1.6	140	13	21606
57	Afar 5	58797	4610	2059	1426	8.0	760	16	67676
58	Afar 6	36116	4560	1836	899	4.4	330	16	43761
59	Afar 7	3599	3751	45	58	0.4	110	16	7580
60	Larderello 1	22013	2156	63	116	1.2	51	29	24429
61	Larderello 2	4146	2278	19	39	4.1	41	10	6536
62	Larderello 3	41521	11029	73	1078	4.5	106	20	53831
63	Larderello 4	26591	12695	91	741	6.2	98	18	40240
64	Larderello 5	44153	14999	82	195	4.7	79	30	59543
65	Deception Island 1	16306	459	17	749	1.5	35	7.3	17575
66	Deception Island 2	14763	516	25	769	1.9	45	7.3	16127
67	Deception Island 3	20023	539	21	836	2.7	65	7.3	21494
68	Copahue volcano 1	1421	319	6.3	142	0.6	26	17	1932
69	Copahue volcano 2	2073	316	9.5	81	0.8	31	13	2524
70	Copahue volcano 3	1825	358	5.8	62	0.9	44	13	2309
71	Copahue volcano 4	7375	449	9.9	120	0.5	43	15	8012
72	Copahue volcano 5	3389	482	9.8	137	0.7	56	9.3	4084
73	Copahue volcano 6	7253	519	12	103	0.6	43	12	7942
74	Copahue volcano 7	427	539	5.1	13	0.8	36	17	1038
75	Copahue volcano 8	789	449	6.7	23	0.9	38	19	1325
76	Copahue volcano 9	2914	462	8.6	51	1.1	55	19	3511
77	Copahue volcano 10	2954	415	7.6	140	0.9	91	22	3631
78	Nysiros Island 2	438	4569	32	32	0.7	11	113	5195
79	Nysiros Island 3	877	2889	7.5	45	2.1	20	86	3927
80	Nysiros Island 4	6905	3396	14	244	0.5	43	102	10704
81	Nysiros Island 5	3012	3836	34	115	2.6	54	126	7180
82	Nysiros Island 6	3396	2684	18	97	1.7	40	67	6305
83	Nysiros Island 7	566	3397	48	39	1.5	12	67	4131
84	Nysiros Island 8	2084	3371	16	51	1.8	18	66	5608
85	Nysiros Island 9	4970	3501	0.6	117	1.6	20	77	8687
86	Nysiros Island 10	748	3215	12	40	1.3	20	66	4103
87	Nysiros Island 11	7957	3056	39	271	1.5	54	136	11515
88	Nysiros Island 12	3089	3589	26	37	1.9	21	82	6846
89	Nysiros Island 13	864	3451	5.3	25	1.6	40	50	4437
90	Nysiros Island 15	675	2916	1.5	15	1.5	20	68	3697
91	Nysiros Island 16	436	2615	1.6	31	2.4	23	66	3174
92	Ischia Island 1	2114	406	4.9	89	2.6	82	5.9	2704
93	Ischia Island 2	1016	361	2.2	45	0.2	46	4.5	1474
94	Ischia Island 4	3833	469	4.1	96	0.5	77	6.8	4487
95	Ischia Island 5	1420	498	3.8	51	1.4	28	5.9	2009
96	Ischia Island 6	2426	422	3.9	69	0.5	92	5.2	3018
97	Phlegrean Fields 1	373	597	4.9	2.2	1.1	33	5.4	1017
98	Phlegrean Fields 2	586	556	3.6	3.1	4.0	66	4.9	1224
99	Phlegrean Fields 3	591	668	3.8	2.7	2.0	48	5.4	1321
100	Phlegrean Fields 4	970	349	3.9	4.9	1.5	32	6.0	1367
101	Phlegrean Fields 5	1804	853	7.9	14	2.3	76	8.1	2765
102	Phlegrean Fields 6	2248	716	7.1	18	2.1	51	8.1	3050
103	Phlegrean Fields 7	1631	384	4.4	8.8	2.1	54	6.8	2091
104	Phlegrean Fields 8	1102	516	2.4	6.6	1.7	36	9.0	1673
105	Phlegrean Fields 9	827	412	1.9	5.4	2.0	25	10	1283
106	Phlegrean Fields 10	642	459	5.1	3.5	2.2	37	6.2	1155
107	Phlegrean Fields 11	2150	334	3.6	15	2.1	33	5.0	2543
108	Vesuvio volcano 1	7897	783	5.6	43	1.4	36	12	8778
109	Vesuvio volcano 2	7599	651	6.1	37	1.6	32	13	8340
110	Pantelleria Island 1	81	196	2.4	4.4	0.1	1.8	4.2	290
111	Pantelleria Island 2	24	148	1.3	2.3	0.1	1.2	3.1	180
112	Pantelleria Island 3	239	186	3.7	10	0.1	5.2	4.3	449
113	El Chichon volcano 1	427	503	11	34	1.6	5.0	52	1034
114	El Chichon volcano 2	9285	1364	55	284	2.7	21	72	11084
115	El Chichon volcano 3	1421	718	13	30	1.0	9.0	70	2262
116	El Chichon volcano 4	1305	611	13	115	1.5	7.0	74	2126
117	El Chichon volcano 5	570	408	10	34	0.3	4.0	37	1064
118	El Chichon volcano 6	309	417	11	18	0.9	1.7	45	802
119	Tatun 1	362759	37219	9557	3150	8.4	2759	282	415733
120	Tatun 2	51563	31045	2400	798	6.0	1093	323	87227
121	Tatun 3	37431	30749	2411	727	3.1	566	85	71973
122	Tatun 4	4078	5195	238	85	0.2	72	5	9674
123	Tatun 5	16862	13048	1519	347	1.9	220	81	32078
124	Tatun 6	7363	593	199	64	0.8	140	1.0	8361
125	Tatun 7	29548	2581	697	362	5.5	783	45	34022
126	Yellowstone 1	339119	3659	4020	2694	26	2274	22	351814
127	Yellowstone 2	121602	8281	3237	4040	21	1599	24	138804
128	Yellowstone 3	174226	16987	1739	4662	49	1387	15	199066
129	Yellowstone 4	190197	18516	3805	2091	65	1561	69	216304
130	Yellowstone 5	150632	8311	1634	1295	28	1275	43	163218
131	Yellowstone 6	258551	10288	3019	2757	29	1767	43	276453
132	Yellowstone 7	489610	9410	10816	4226	75	3605	40	517782
133	Yellowstone 8	79168	2164	1327	2274	35	639	35	85641
134	Yellowstone 9	320241	3399	1987	3556	28	1250	35	330496
135	Yellowstone 10	40923	1187	871	1296	49	356	43	44725
136	Yellowstone 11	1012181	8996	9685	9178	78	6698	27	1046843
137	Yellowstone 12	47727	671	560	1313	20	210	21	50522
138	Yellowstone 13	413725	5214	9650	4224	66	1818	41	434738
139	Yellowstone 14	1156785	27956	34552	9520	76	6999	54	1235942

**Table 3.** Composition of heteroaromatics, C_6_H_6_ and C_7_H_8_. Concentrations are in ppbv.

**n°**	**sample**	**C**_**4**_**H**_**4**_**O**	**2-C**_**5**_**H**_**6**_**O**	**C**_**4**_**H**_**8**_**O**	**3-C**_**5**_**H**_**10**_**O**	**C**_**4**_**H**_**4**_**S**	**3-C**_**5**_**H**_**6**_**S**	**2,4-C**_**6**_**H**_**8**_**S**	**C**_**6**_**H**_**6**_	**C**_**7**_**H**_**8**_
1	Teide volcano 1	1.3	0.6	0.3	<0.1	12	1.8	<0.1	71	0.5
2	Teide volcano 2	1.6	0.7	0.4	<0.1	11	1.7	<0.1	89	0.5
3	Lascar volcano 3	16	1.9	4.5	0.9	2.6	0.7	<0.1	21	0.8
4	Lascar volcano 4	12	1.5	6.3	0.9	3.1	0.5	<0.1	19	0.7
5	Lascar volcano 5	11	1.8	7.8	1.2	4.5	0.6	<0.1	23	0.8
6	Lascar volcano 6	13	1.4	6.3	0.8	3.9	0.3	<0.1	26	0.8
7	Lascar volcano 7	10	1.3	5.6	0.9	6.3	0.5	<0.1	18	0.7
8	Lascar volcano 8	12	1.9	7.8	0.6	7.2	0.4	<0.1	17	0.7
9	Lascar volcano 9	14	1.5	7.5	0.7	1.9	0.3	<0.1	13	0.6
10	Lascar volcano 10	12	1.3	6.3	0.9	1.8	0.2	<0.1	14	0.8
11	Lascar volcano 11	13	1.7	5.7	0.7	1.6	0.3	<0.1	12	0.6
12	Lascar volcano 12	12	1.6	5.4	0.5	1.8	0.1	<0.1	12	0.7
13	Lascar volcano 13	13	1.2	4.6	0.5	1.5	0.3	<0.1	13	0.7
14	Lascar volcano 14	10	1.3	5.1	0.6	1.6	0.2	<0.1	14	0.5
15	Tacora volcano 1	1.2	0.4	0.5	<0.1	15	1.6	0.2	238	5.8
16	Tacora volcano 2	0.8	0.6	0.4	0.1	18	1.9	0.1	274	7.6
17	Tacora volcano 3	1.3	0.8	0.4	<0.1	16	1.3	0.1	243	5.8
18	Tacora volcano 4	1.4	0.7	0.6	0.2	24	2.1	0.1	96	4.4
19	Tacora volcano 5	1.6	0.9	0.6	0.3	12	2.2	0.2	116	7.6
20	Tacora volcano 6	1.3	0.6	0.4	<0.1	16	1.9	0.1	132	6.7
21	Tacora volcano 7	1.4	0.6	0.8	0.2	13	1.6	0.2	155	7.1
22	Tacora volcano 8	1.3	0.7	0.5	<0.1	10	1.3	0.3	152	6.2
23	Tacora volcano 9	1.4	0.8	0.4	0.1	10	1.5	0.2	168	7.1
24	Tacora volcano 10	1.1	0.4	0.6	<0.1	11	1.8	0.2	164	7.6
25	Turrialba volcano 5	4.4	0.6	1.7	0.8	5.7	0.1	<0.1	9.1	0.3
26	Turrialba volcano 6	4.4	0.8	2.3	0.7	6.2	0.3	<0.1	8.8	0.1
27	Turrialba volcano 7	6.3	0.7	2.1	0.6	2.6	0.2	<0.1	8.7	0.2
28	Turrialba volcano 8	7.1	0.9	1.6	0.8	1.1	0.1	<0.1	10	0.2
29	Vulcano Island crater 1	16	2.2	4.8	1.5	0.4	0.1	<0.1	1.5	0.2
30	Vulcano Island crater 2	10	1.5	5.5	1.3	0.7	0.1	<0.1	6.0	0.3
31	Vulcano Island crater 3	23	2.3	4.4	1.5	0.5	<0.1	<0.1	2.5	0.1
32	Vulcano Island crater 4	14	1.2	5.2	1.8	0.2	<0.1	<0.1	1.5	<0.1
33	Vulcano Island crater 5	16	1.5	5.6	1.9	0.2	<0.1	<0.1	1.2	<0.1
34	Vulcano Island crater 6	13	1.4	0.8	0.2	0.5	0.3	<0.1	1.2	<0.1
35	Vulcano Island crater 7	19	2.3	6.3	1.7	0.2	<0.1	<0.1	1.6	<0.1
36	Vulcano Island crater 8	4.7	0.6	0.7	0.2	1.9	0.2	<0.1	18	0.5
37	Vulcano Island crater 9	25	3.3	3.8	1.5	0.6	<0.1	<0.1	1.4	<0.1
38	Vulcano Island crater 10	15	1.2	5.2	1.5	0.4	<0.1	<0.1	2.3	0.1
39	Vulcano Island crater 11	31	3.1	4.1	1.8	0.2	<0.1	<0.1	1.7	<0.1
40	Vulcano Island crater 12	14	1.1	3.7	1.6	0.3	<0.1	<0.1	3.0	0.1
41	Vulcano Island crater 13	16	1.3	5.2	1.8	0.2	<0.1	<0.1	1.0	0.1
42	Vulcano Island crater 14	17	1.2	5.8	1.7	0.3	<0.1	<0.1	3.1	<0.1
43	Vulcano Island crater 15	21	2.0	4.6	1.4	0.2	<0.1	<0.1	1.5	0.1
44	Vulcano Island crater 16	26	2.7	4.5	1.6	0.5	0.1	<0.1	7.0	0.3
45	Vulcano Island crater 17	2.2	0.5	0.6	0.3	1.5	0.2	<0.1	9.8	0.5
46	Vulcano Island crater 18	4.5	0.9	3.2	1.2	0.4	<0.1	<0.1	14	0.4
47	Vulcano Island crater 19	15	1.8	3.9	1.3	0.3	<0.1	<0.1	1.2	0.1
48	El Tatio 1	<0.1	<0.1	<0.1	<0.1	1.5	1.1	0.6	673	180
49	El Tatio 2	<0.1	<0.1	<0.1	<0.1	1.5	1.6	0.6	1009	657
50	El Tatio 3	<0.1	<0.1	<0.1	<0.1	1.4	1.7	0.7	425	86
51	El Tatio 4	<0.1	<0.1	<0.1	<0.1	1.1	1.1	0.5	520	181
52	El Tatio 5	<0.1	<0.1	<0.1	<0.1	0.7	1.2	0.4	494	95
53	Afar 1	<0.1	<0.1	<0.1	<0.1	5.6	4.1	1.6	2887	45.5
54	Afar 2	<0.1	<0.1	<0.1	<0.1	6.1	8.7	0.9	2698	75
55	Afar 3	<0.1	<0.1	<0.1	<0.1	7.3	6.3	1.3	3324	180
56	Afar 4	<0.1	<0.1	<0.1	<0.1	5.9	6.1	0.9	4102	360
57	Afar 5	<0.1	<0.1	<0.1	<0.1	6.6	7.4	2.1	3845	725
58	Afar 6	<0.1	<0.1	<0.1	<0.1	6.1	8.6	1.6	4175	365
59	Afar 7	<0.1	<0.1	<0.1	<0.1	5.5	9.1	1.8	3702	42.0
60	Larderello 1	<0.1	<0.1	<0.1	<0.1	11	12	5.5	1274	873
61	Larderello 2	<0.1	<0.1	<0.1	<0.1	3.2	4.4	2.1	1368	899
62	Larderello 3	<0.1	<0.1	<0.1	<0.1	10	5.6	4.3	6888	4129
63	Larderello 4	<0.1	<0.1	<0.1	<0.1	8	6.9	3.4	8409	4276
64	Larderello 5	<0.1	<0.1	<0.1	<0.1	12	13	4.7	12298	2660
65	Deception Island 1	0.3	0.6	0.4	<0.1	2.8	2.1	1.1	351	99
66	Deception Island 2	0.2	0.7	0.5	<0.1	2.9	2.2	0.8	415	84
67	Deception Island 3	0.3	0.6	0.4	<0.1	2.9	2.3	0.8	426	101
68	Copahue volcano 1	0.2	0.8	1.9	0.8	5.1	5.6	2.6	189	120
69	Copahue volcano 2	0.1	0.4	1.1	0.8	4.3	4.1	2.4	256	46
70	Copahue volcano 3	0.3	0.6	1.7	0.6	3.8	3.5	2.5	289	55
71	Copahue volcano 4	0.2	0.7	2.1	0.7	3.6	3.9	3.6	377	65
72	Copahue volcano 5	0.3	0.6	1.5	0.9	2.1	2.5	1.4	449	30
73	Copahue volcano 6	0.3	0.8	1.2	0.5	3.9	3.4	1.6	487	26
74	Copahue volcano 7	0.2	0.5	1.7	0.8	4.9	5.6	3.2	520	11
75	Copahue volcano 8	0.4	0.6	1.1	0.4	6.1	5.2	4.8	426	11
76	Copahue volcano 9	0.3	0.4	1.0	0.5	5.6	4.9	6.1	441	13
77	Copahue volcano 10	0.3	0.7	0.9	0.4	6.2	6.6	6.9	389	12
78	Nysiros Island 2	0.2	0.1	0.6	<0.1	61	46	5.6	4260	295
79	Nysiros Island 3	0.2	0.1	0.5	<0.1	36	44	6.2	2750	125
80	Nysiros Island 4	0.3	0.2	0.8	<0.1	44	51	7.4	3159	230
81	Nysiros Island 5	0.1	<0.1	0.4	<0.1	53	67	5.9	3715	110
82	Nysiros Island 6	0.1	<0.1	0.3	<0.1	37	25	5.4	2518	155
83	Nysiros Island 7	<0.1	<0.1	0.4	<0.1	39	22	6.2	3196	185
84	Nysiros Island 8	0.2	0.1	0.5	<0.1	41	20	4.8	3239	110
85	Nysiros Island 9	<0.1	<0.1	0.4	<0.1	42	29	5.9	3291	180
86	Nysiros Island 10	<0.1	<0.1	0.6	<0.1	44	18	4.2	3102	95
87	Nysiros Island 11	0.2	0.1	0.7	<0.1	58	74	3.8	2881	130
88	Nysiros Island 12	0.3	0.1	0.7	<0.1	43	33	6.1	3470	105
89	Nysiros Island 13	0.3	0.2	0.9	<0.1	30	13	7.2	3248	185
90	Nysiros Island 15	<0.1	<0.1	0.4	<0.1	36	26	6.2	2735	160
91	Nysiros Island 16	<0.1	<0.1	0.3	<0.1	37	22	6.6	2469	125
92	Ischia Island 1	<0.1	<0.1	<0.1	<0.1	2.3	2.8	0.8	357	39
93	Ischia Island 2	<0.1	<0.1	<0.1	<0.1	2.1	1.5	0.9	323	34
94	Ischia Island 4	<0.1	<0.1	<0.1	<0.1	2.6	3.3	0.9	392	65
95	Ischia Island 5	<0.1	<0.1	<0.1	<0.1	3.1	2.3	0.5	452	38
96	Ischia Island 6	<0.1	<0.1	<0.1	<0.1	2.1	2.5	0.6	364	48
97	Phlegrean Fields 1	<0.1	<0.1	<0.1	<0.1	3.3	1.5	0.6	573	12
98	Phlegrean Fields 2	<0.1	<0.1	0.2	<0.1	2.8	1.6	0.5	507	42
99	Phlegrean Fields 3	<0.1	<0.1	<0.1	<0.1	2.1	2.6	0.7	609	47
100	Phlegrean Fields 4	<0.1	<0.1	0.1	<0.1	2.6	2.6	0.8	304	39
101	Phlegrean Fields 5	<0.1	<0.1	0.1	<0.1	3.4	3.9	0.8	762	84
102	Phlegrean Fields 6	<0.1	<0.1	<0.1	<0.1	3.1	4.1	0.9	579	130
103	Phlegrean Fields 7	<0.1	<0.1	0.2	<0.1	2.6	3.6	0.6	567	110
104	Phlegrean Fields 8	<0.1	<0.1	<0.1	<0.1	3.5	4.6	0.9	408	90
105	Phlegrean Fields 9	<0.1	<0.1	<0.1	<0.1	4.1	5.1	0.9	322	75
106	Phlegrean Fields 10	<0.1	<0.1	<0.1	<0.1	2.4	3.1	0.7	364	85
107	Phlegrean Fields 11	<0.1	<0.1	<0.1	<0.1	2.0	2.4	0.6	303	23
108	Vesuvio volcano 1	0.2	0.1	0.4	<0.1	5.6	5.9	0.5	763	13
109	Vesuvio volcano 2	0.2	0.1	0.3	<0.1	5.8	6.1	0.7	632	9.8
110	Pantelleria Island 1	<0.1	<0.1	<0.1	<0.1	1.9	1.5	0.8	135	48
111	Pantelleria Island 2	<0.1	<0.1	<0.1	<0.1	1.5	1.1	0.5	95	44
112	Pantelleria Island 3	<0.1	<0.1	<0.1	<0.1	2.3	1.4	0.6	129	43
113	El Chichon volcano 1	0.3	0.7	1.6	0.8	11	36	1.5	416	75
114	El Chichon volcano 2	0.4	0.5	1.5	0.6	12	56	1.3	607	750
115	El Chichon volcano 3	0.2	0.4	2.2	0.7	11	54	1.5	317	390
116	El Chichon volcano 4	0.2	0.6	1.4	0.5	10	59	2.2	269	325
117	El Chichon volcano 5	0.3	0.8	1.8	0.5	7.8	26	0.3	332	60
118	El Chichon volcano 6	0.3	0.9	1.6	0.7	10	31	0.4	337	70
119	Tatun 1	<0.1	<0.1	<0.1	<0.1	156	113	13	26522	10650
120	Tatun 2	<0.1	<0.1	<0.1	<0.1	191	121	11	23048	7800
121	Tatun 3	<0.1	<0.1	<0.1	<0.1	54	28	2.9	21579	9050
122	Tatun 4	<0.1	<0.1	<0.1	<0.1	2.3	2.1	0.3	3871	1250
123	Tatun 5	<0.1	<0.1	<0.1	<0.1	42	36	2.5	9696	3350
124	Tatun 6	<0.1	<0.1	<0.1	<0.1	0.6	0.3	0.1	407	180
125	Tatun 7	<0.1	<0.1	<0.1	<0.1	24	20	1.0	1929	600
126	Yellowstone 1	<0.1	<0.1	<0.1	<0.1	10	12	0.6	2999	625
127	Yellowstone 2	<0.1	<0.1	<0.1	<0.1	11	13	0.4	6602	1655
128	Yellowstone 3	<0.1	<0.1	<0.1	<0.1	8.7	6.1	0.3	13615	3295
129	Yellowstone 4	<0.1	<0.1	<0.1	<0.1	31	36	1.5	14909	3555
130	Yellowstone 5	<0.1	<0.1	<0.1	<0.1	19	23	0.8	6600	1680
131	Yellowstone 6	<0.1	<0.1	<0.1	<0.1	16	26	1.1	9382	870
132	Yellowstone 7	<0.1	<0.1	<0.1	<0.1	15	24	1.2	7590	1770
133	Yellowstone 8	<0.1	<0.1	<0.1	<0.1	18	16	0.6	1573	560
134	Yellowstone 9	<0.1	<0.1	<0.1	<0.1	14	21	0.5	2435	925
135	Yellowstone 10	<0.1	<0.1	<0.1	<0.1	16	26	1.1	816	325
136	Yellowstone 11	<0.1	<0.1	<0.1	<0.1	10	16	0.5	7795	1155
137	Yellowstone 12	<0.1	<0.1	<0.1	<0.1	8.9	12	0.3	532	125
138	Yellowstone 13	<0.1	<0.1	<0.1	<0.1	19	21	1.2	3260	1930
139	Yellowstone 14	<0.1	<0.1	<0.1	<0.1	24	29	1.1	23311	4575
